# Beyond algorithmic trust: human–AI interaction competency strengthens AI-driven financial decision-making and financial resilience

**DOI:** 10.3389/frai.2026.1867266

**Published:** 2026-06-01

**Authors:** M. Vijayananth, N. Saravanabhavan

**Affiliations:** Department of Commerce, Vellore Institute of Technology, Vellore, Tamil Nadu, India

**Keywords:** AI-driven trading, algorithmic trust, financial resilience, human–AI interaction competency, S-O-R model, sustainable investment

## Abstract

**Introduction:**

This study examines the impact of AI trading usage on investor financial resilience through a cognitive-behavioral process grounded in the Stimulus-Organism-Response (S-O-R) framework.

**Methods:**

AI trading usage and perceived financial uncertainty were conceptualized as stimuli, perceived algorithmic trust as the organism, and sustainable investment behavior and investor financial resilience as sequential responses. Data were collected from 569 middle-income retail investors across major Indian cities and analyzed using Partial Least Squares Structural Equation Modeling (PLS-SEM).

**Results:**

AI trading usage and perceived financial uncertainty positively influenced perceived algorithmic trust, which subsequently enhanced sustainable investment behavior and investor financial resilience. Human–AI interaction competency significantly moderated the relationship between perceived algorithmic trust and sustainable investment behavior.

**Discussion:**

The study advances understanding of AI-driven financial decision-making by highlighting the sequential role of perceived algorithmic trust, sustainable investment behavior, and Human–AI interaction competency in strengthening investor financial resilience and adaptive investment practices.

## 1 Introduction

The rapid integration of artificial intelligence (AI) into the financial market is radically changing the way investors acquire information, evaluate risk, and make decisions. Algorithmic trading systems, robo-advisory services, and predictive analytics are some of the technologies that make trading more efficient, analytical, and accessible to retail investors (Gomber et al., 2017; Dwivedi et al., 2021; Flavián et al., 2022). Those technologies have become the centre of a new financial ecosystem by allowing real-time analysis of massive financial data, which shapes investment behavior and performance (Lee and Shin, 2018; Huang and Rust, 2021).

The growing adoption of AI in finance has also accelerated the emergence of intelligent investment ecosystems, where machine learning algorithms, predictive analytics, and AI-assisted advisory systems increasingly support investor cognition and financial decision-making (Kshetri et al., 2024; Asemi et al., 2025; Aldasoro et al., 2025). Such systems are transforming the interaction between human investors and financial technologies by enabling automated interpretation of market signals, personalized investment recommendations, and real-time risk evaluation (Xu and Cho, 2025). Consequently, understanding how investors cognitively respond to AI-generated financial insights has become an important research concern within AI-enabled financial services.

Meanwhile, sustainability has emerged as one of the major factors to be considered in making investment decisions. Investors are increasingly incorporating environmental, social, and governance (ESG) considerations in order to attain long-term value and responsible results (Friede et al., 2015; Fatemi et al., 2018; Berg et al., 2022). AI-driven tools can support this transition, as they allow evaluating ESG-related information efficiently and detect sustainability risks and opportunities (Eccles et al., 2020; Krueger et al., 2020). Sustainable investment behavior has thus emerged as a major aspect of the modern-day investment practices (Broadstock et al., 2021).

In spite of these developments, the use of AI-driven systems poses issues of transparency and trust (Rai, 2020; Shin, 2021; Hoang et al., 2026). Most of the models are opaque algorithmic systems that do not provide insight to users on how recommendations are generated (Dietvorst et al., 2015; Logg et al., 2019). This interpretability can diminish trust and decision-making, resulting in over-dependence or unwillingness to use such tools. In this context, perceived algorithmic trust emerges as a decisive factor influencing the acceptance and use of AI-generated financial insights, consistent with the Technology Acceptance Model (TAM) and Trust Theory, in which perceived usefulness and reliability determine trust in technology.

These challenges become more pronounced under conditions of financial uncertainty, where market volatility and informational asymmetry increase the complexity of investment decision-making (Baker et al., 2016; Jurado et al., 2015). Under these circumstances, the market participants rely more on the AI systems to decode signals and risk management. Nevertheless, making effective decisions is not just a matter of technological capacity, but also the cognitive abilities of individuals to process and use algorithmic knowledge. Uncertainty increases the sensitivity of losses as postulated by the Prospect Theory, which affects the use of decision aids. This highlights the necessity to explore the mechanisms that connect AI use to investment behavior and financial performance.

The present study addresses this issue by applying the Stimulus-Organism-Response (SOR) model. This way, the AI trading usage and the perceived financial uncertainty are seen as stimuli that influence investment decision-making. These affect the internal cognitive behavioral processes such as perceived algorithmic trust, which can lead to sustainable investment behavior.

Sustainable investment behavior is conceptualised as an internal process of behavioral orientation, rather than immediate response that needs to be in line with the organism stage.

Within this process, perceived algorithmic trust functions as a key cognitive mechanism that transforms AI-generated stimuli into sustainable investment behavior. In turn, sustainable investment behavior leads to investor financial resilience, which can be understood as the ability to survive market changes, recover from market shocks and establish long-term financial stability (Salignac et al., 2019). This behavior helps to tackle uncertainty, by promoting a sustained and disciplined investment approach.

The framework also adds Human–AI interaction competency as a moderating variable between perceived algorithmic trust and sustainable investment behavior, especially as a driver to convert cognitive trust into actionable investment behavior. This shows the assumption that trust alone is not sufficient to motivate behavior: people should have the ability to read and apply AI-generated information.

Even though earlier studies have explored the adoption of AI, sustainable investing, and financial behavior, these fields are not yet unified (Flavián et al., 2022; Kshetri et al., 2024). The sequential mechanism connecting AI usage, trust, sustainable behavior, and resilience is rarely modeled in the study conducted in the past, as well as the boundary condition of Human–AI interaction competency, especially in the context of emerging markets with financial uncertainty.

To address this gap, the study develops a unified framework explaining how AI-driven trading influences investor financial resilience through perceived algorithmic trust and sustainable investment behavior, with human–AI interaction competency as a moderating factor. The study focuses on middle-income retail investors in Indian metropolitan cities.

Accordingly, the study addresses the following research questions:

*RQ1*: How do AI trading usage and perceived financial uncertainty influence perceived algorithmic trust?

*RQ2*: How does perceived algorithmic trust shape sustainable investment behavior?

*RQ3*: How does sustainable investment behavior influence investor financial resilience?

*RQ4*: How does human–AI interaction competency moderate the relationship between perceived algorithmic trust and sustainable investment behavior?

This study contributes by introducing a sequential S-O-R-based mechanism and identifying human–AI interaction competency as a key boundary condition in AI-enabled financial decision-making.

The rest of this paper is organized in the following way. Section 2 discusses the literature and formulates the hypotheses. Section 3 discusses the research design based on PLS-SEM. The empirical findings are reported in section 4. Section 5 addresses the findings and conclusions, limitations and future research directions. Section 6 summarizes the study.

## 2 Literature review and hypothesis development

### 2.1 Theoretical foundation

This study is based on the Stimulus Organism Response (SOR) model, which describes the effects of external stimuli on behavior via internal cognitive processes (Mehrabian and Russell, 1974; Jacoby, 2002). The model assumes that the external stimuli influence the organismic states, which in turn affect behavioral responses (Eroglu et al., 2001). Recent studies prove its applicability to the technology-driven context, especially in the description of user behavior in digital and AI-driven environments (Jang et al., 2021).

In the current research, AI trading usage and perceived financial uncertainty function serve as important stimulus factors affecting investor decision-making (Baker et al., 2016; Dwivedi et al., 2021). These aspects influence the organismic state, represented by the perceived algorithmic trust, which promotes sustainable investment behavior (ESG-oriented) as an internalized behavioral orientation facilitated by AI-enhanced ESG information processing (Glikson and Woolley, 2020; Berg et al., 2022). The resulting response is investor financial resilience, reflecting the ability to adapt to volatility and remain financially stable (Salignac et al., 2019).

To strengthen this framework, the study integrates complementary theories. Technology Acceptance Model describes the effect of perceived usefulness on the adoption of technology and the development of trust (Davis, 1989; Venkatesh and Davis, 2000) and Trust Theory highlights the role of trust in reducing uncertainty and making it possible to rely on complex systems (Mayer et al., 1995; McKnight et al., 2002). The empirical evidence also supports the core idea that trust is a key factor in adopting AI and decision-making in the digital environment (Glikson and Woolley, 2020; Choung et al., 2023).

Furthermore, Prospect Theory describes how people react to uncertainty by being loss-averse and risk-perceptive (Tversky and Kahneman, 1992; Kahneman and Tversky, 2013). In the circumstances of financial uncertainty, people tend to follow decision aids like AI systems to minimize ambiguity and enhance the quality of decisions (Jurado et al., 2015; Baker et al., 2016). This supports the importance of perceived financial uncertainty as a powerful stimulus affecting cognitive evaluations.

Synthesising these perspectives, the study proposes a cognitive-behavioral, sequential model in which investors’ AI trading usage and perceived financial uncertainty drive their perceived algorithmic trust, which then drives sustainable investment behavior and investor financial resilience.

### 2.2 AI trading usage, financial uncertainty and algorithmic trust

AI trading usage indicates the degree to which investors use algorithmic systems to analyze the market, make predictions, and support their decisions. These systems boost information processing and decrease mental effort, which leads to greater acceptability of algorithmic suggestions (Dwivedi et al., 2021; Choung et al., 2023).

The further use of these tools reinforces the feelings of reliability and usefulness, which are key factors in trust development (Logg et al., 2019; Glikson and Woolley, 2020). In financial settings, this experience promotes trust in algorithmic trading decisions. Therefore, higher AI trading usage is expected to enhance perceived algorithmic trust.

Perceived financial uncertainty refers to investors’ perceptions of uncertainty about market conditions that create more ambiguity and complexity in decision-making (Jurado et al., 2015; Baker et al., 2016). In these circumstances, people may be more likely to use rigid, data-based decision-making systems to process market information and simplify their decision-making.

Existing behavioral studies indicates that uncertainty may lead to greater reliance on algorithmic decision support systems, because of their perceived stability and problem-solving ability (Logg et al., 2019; Kahneman and Tversky, 2013). In financial markets, this could lead to increased confidence in algorithmic suggestions, thereby increasing perceived algorithmic trust.

Accordingly, the following hypotheses are proposed:

*H1a*: AI trading usage positively influences perceived algorithmic trust.

*H1b*: Perceived financial uncertainty positively influences perceived algorithmic trust.

### 2.3 Perceived algorithmic trust, sustainable investment behavior, and investor financial resilience

Perceived algorithmic trust refers to investors’ belief in the accuracy and effectiveness of AI-based decision-support algorithms (Glikson and Woolley, 2020; Choung et al., 2023). In financial contexts, it lowers perceived risk and promotes the use of data-driven recommendations, which in turn promote systematic and disciplined financial decision-making (Logg et al., 2019; Dietvorst et al., 2015). Perceived algorithmic trust as an organismic state affects investors’ behavioral tendencies by allowing them to include systematic and data-driven assessments in their decision-making. Therefore, perceived algorithmic trust enhances sustainable investment behavior.

*H2*: Perceived algorithmic trust positively influences sustainable investment behavior.

Furthermore, sustainable investment behavior reflects a disciplined investment strategy that considers sustainable values and risk factors in financial decisions (Friede et al., 2015; Broadstock et al., 2021). This investment mindset helps improve investor financial resilience, which refers to the capacity to manage financial shocks, adapt to market conditions and maintain financial stability (Salignac et al., 2019). Existing studies reveals that sustainable investment practices offer higher stability and protection against downside risk in uncertain market conditions (Albuquerque et al., 2020; Broadstock et al., 2021). As a result, sustainable investment behavior enhances investor financial resilience, representing the response outcome of the framework.

*H3*: Sustainable investment behavior positively influences investor financial resilience.

### 2.4 Mediating effects of perceived algorithmic trust and sustainable investment behavior

Mediation refers to the internal processes that transmit the influence of external factors on behavior. Perceived algorithmic trust and sustainable investment behavior are the main conduits in this research that carry the impact of AI trading usage and perceived financial uncertainty to investor financial outcomes.

Investors perceived algorithmic trust is the primary process used to interpret AI information. Trust in intelligent systems is likely to decrease perceived uncertainty and increase the usage of data-driven insights, thus facilitating the translation of stimuli into actions (Glikson and Woolley, 2020; Choung et al., 2023). Other research also indicates that trust in digital technologies facilitates the use of technology-based insights (Siau and Wang, 2018; Cao et al., 2021). As such, the use of AI trading and perceived financial uncertainty is likely to affect sustainable investment behavior indirectly through perceived algorithmic trust.

*H4a*: Perceived algorithmic trust mediates the relationship between AI trading usage and sustainable investment behavior.

*H4b*: Perceived algorithmic trust mediates the relationship between perceived financial uncertainty and sustainable investment behavior.

Furthermore, sustainable investment behavior is a key behavioral pathway that translates cognitive assessments into long-term financial results. These practices encourage disciplined investment strategies and risk management, which contribute to increased stability in uncertain environments (Broadstock et al., 2021; Gillan et al., 2021). As a result, perceived algorithmic trust is expected to impact investor financial resilience via sustainable investment behavior.

*H5*: Sustainable investment behavior mediates the relationship between perceived algorithmic trust and investor financial resilience.

### 2.5 Sequential mediation of perceived algorithmic trust and sustainable investment behavior

Sequential mediation describes how multiple internal processes combine to convey the effects of external factors to outcomes. In this research, perceived algorithmic trust and sustainable investment behavior function as sequential processes that bridge the impact of AI trading usage and perceived financial uncertainty on investor financial resilience.

Perceived algorithmic trust is the first cognitive process in which investors assess AI-based information, leading to greater trust in data-driven technologies (Glikson and Woolley, 2020; Choung et al., 2023). This perception then translates into sustainable investment behavior, where investors adopt structured and long-term considerations in their investment decisions (Berg et al., 2022; Kshetri et al., 2024). These cognitive stages represent a dual pathway through which external influences are translated into adaptive investment practices.

Ultimately, sustainable investment behavior leads to investor financial resilience, which is the capacity to absorb financial shocks and maintain financial stability (Broadstock et al., 2021; Gillan et al., 2021). This sequential pathway highlights how trust formation and behavioral adjustment jointly transmit the influence of external factors to final outcomes.

Accordingly, the following hypotheses are proposed:

*H6a*: Perceived algorithmic trust and sustainable investment behavior sequentially mediate the relationship between AI trading usage and investor financial resilience.

*H6b*: Perceived algorithmic trust and sustainable investment behavior sequentially mediate the relationship between perceived financial uncertainty and investor financial resilience.

### 2.6 Moderating role of human–AI interaction competency

Human–AI interaction competency is defined as an investor’s capacity to understand and apply insights derived from AI systems (Logg et al., 2019; Choung et al., 2023). This competency encompasses both technological awareness and the ability to interpret algorithmic insights in financial contexts. Existing studies suggest that people with greater technological competence make greater use of sophisticated analytical systems, which in turn leads to better decision-making and more trust in data-driven processes (Dwivedi et al., 2021).

Human–AI interaction competency serves as a moderator that influences the translation from cognitive to actionable outcomes. Specifically, the effect of perceived algorithmic trust on sustainable investment behavior depends on an investor’s ability to engage with AI systems. Investors with greater competency are more likely to understand algorithmic insights and use them appropriately, which enhances the impact of trust on ESG investment decisions (Choung et al., 2023).

Thus, this study proposes the following hypothesis:

*H7*: Human–AI interaction competency positively moderates the relationship between perceived algorithmic trust and sustainable investment behavior.

### 2.7 Conceptual framework

This research presents a conceptual model that illustrates the impact of AI-based trading on investor financial resilience through a sequence of cognitive and behavioral responses. Drawing on the Stimulus-Organism-Response (SOR) model, the framework explains how external stimuli contribute to adaptive investor responses in the context of AI trading.

AI trading usage and perceived financial uncertainty are conceptualised as external stimuli that influence investor behavior. These factors influence the organismic state, represented by perceived algorithmic trust, which reflects investors’ confidence in data-driven decision-support systems. Trust is a key cognitive process that allows investors to understand and use algorithmic insights.

In turn, perceived algorithmic trust leads to sustainable investment behavior, capturing a long-term and disciplined investment approach based on insights. Such investment practice supports investor financial resilience, which is the response outcome that reflects the capacity to manage financial shocks and remain stable during uncertain market conditions.

**Figure 1:**
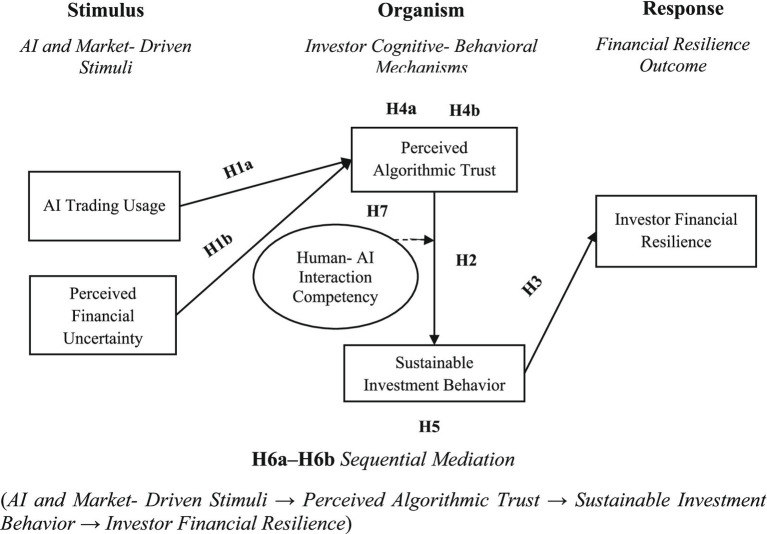
Conceptual framework of the AI-driven investment behavior model under financial uncertainty.

The framework further incorporates a sequential mediation mechanism, whereby perceived algorithmic trust and sustainable investment behavior jointly transmit the effects of external stimuli to investor financial resilience. Moreover, Human–AI interaction competency is proposed as a moderator, which amplifies the link between perceived algorithmic trust and sustainable investment behavior, underscoring the significance of investor competency in the effective use of the AI insights. The study’s conceptual framework is illustrated in Figure 1.

## 3 Methodology

This research uses a quantitative approach to test the proposed conceptual model. The data were collected via a questionnaire survey from middle-income retail investors in India. The proposed hypotheses were tested using PLS-SEM, because it is a suitable approach to test complex models with mediation and moderation and non-normally distributed data (Hair et al., 2019; Sarstedt et al., 2021). The steps followed in sample selection, measurement of constructs, questionnaire development and data analysis are detailed in the subsections below.

### 3.1 Sample and sampling technique

We investigate the attitudes of middle-income retail investors who are increasingly important because they are increasingly involved in stock exchanges and face risks in emerging markets. In this research, these investors are defined as investors who are actively trading on equity markets and are also included in certain income categories as mentioned in the PRICE Survey Report (People Research on India’s Consumer Economy (PRICE), 2022).

As a sampling frame is unavailable to access this population, we used purposive non-probability sampling (Etikan et al., 2016). This approach is widely used to study financial behavior among special groups of investors and is ideal for reaching financial active investors who are otherwise difficult to access through conventional sampling. The questionnaire was distributed via an online survey platform to investor groups, forums and other networks to achieve the desired sample size.

To ensure the representativeness of the sample, we used three criteria: (i) age above 18 years, (ii) middle-income class (as defined in the PRICE report) and (iii) being actively engaged in stock market investments. Only participants who satisfied the three criteria were selected for the study. In January–March 2026, 1,156 surveys were sent via Google Forms. We had received 654 responses (response rate 56.6%). Following data screening for incomplete data, unusual response patterns and screening validation, 85 of them were removed. Thus, 569 responses were used for the final analysis.

The sample size is above the recommended minimum for PLS-SEM analysis, providing sufficient statistical power and precise estimates for the parameters (Hair et al., 2019). The sample comprised professionals from major cities in India - Mumbai, Chennai, Kolkata, Delhi, Bengaluru and Hyderabad. The respondents were informed of the nature of the study, and it was voluntary and anonymous. Prior to data collection, the participants were briefed and consent was sought, and no personal information was collected.

### 3.2 Operationalisation of constructs and survey design

We used multi-item scales from prior studies to measure all the constructs in this study (see Table 1). The variables are measured using the Stimulus-Organism-Response (SOR) model where variables are classified into stimulus, organism and response variables, and a moderating variable (Mehrabian and Russell, 1974; Jacoby, 2002).

**Table 1:** Operationalization of constructs.

Underlying theory	Construct	Measurement indicators	Sources
S-O-R model (stimulus)	AI trading usage (ATU)	Use of AI tools; reliance on algorithmic recommendations; AI-based market analysis; faster decisions using AI	Venkatesh et al. (2012)
S-O-R model (Stimulus)	Perceived financial uncertainty (PFU)	Market unpredictability; difficulty in forecasting; decision uncertainty; perceived instability	Haritha and Uchil (2020)
S-O-R model (organism)	Perceived algorithmic trust (PAT)	Trust in AI recommendations; reliability of outputs; confidence in insights; perceived accuracy	Shin (2021)
S-O-R model (organism)	Sustainable investment behavior (SIB)	ESG consideration; preference for responsible firms; avoidance of unethical investments; long-term focus	Zhang et al. (2024)
S-O-R model (response)	Investor financial resilience (IFR)	Managing downturns; financial stability; handling shocks; recovery from losses	Holmlund et al. (2017)
S-O-R model (moderator)	Human–AI interaction competency (HAI)	Understanding AI outputs; interpreting recommendations; comfort using AI; evaluating usefulness	Xiao and O’Neill (2016); Schepman and Rodway (2020)

AI trading practices (4 items) and perceived financial uncertainty (4 items) are stimuli variables, which are external causes of investor responses. The organism variables are perceived algorithmic trust (4 items) and sustainable investment behavior (4 items), which are internal psychological and behavioral responses of investors. Investor financial resilience (4 items) is the response variable, which reflects the ability to cope with financial adversities and financial well-being. Also, Human–AI interaction competency (4 items) is taken as a moderator that reflects the skills in Human–AI interactions.

Table 1 shows the operationalization of the constructs, measurement items and sources. All measurement items are listed in Appendix Table A1.

**Table A1:** Questionnaire construct, measurement, and sources.

Construct	Items	Measurement	Sources
AI trading usage	ATU 1	I regularly use AI-based tools to support my trading decisions.	Venkatesh et al. (2012)
	ATU 2	I rely on AI platforms for analyzing market trends	
	ATU 3	AI tools help me make faster investment decisions	
	ATU 4	I use algorithm-based recommendations while trading	
Perceived financial uncertainty	PFU 1	I feel uncertain about future market movements	Haritha and Uchil (2020)
	PFU 2	Market conditions are difficult to predict	
	PFU 3	I find it hard to make decisions due to market uncertainty	
	PFU 4	Financial markets often appear unstable to me	
Perceived algorithmic trust	PAT 1	I trust the recommendations provided by AI systems	Shin (2021)
	PAT 2	AI-based tools provide reliable investment advice	
	PAT 3	I feel confident using AI-generated insights for trading	
	PAT 4	AI systems make accurate predictions about the market	
Sustainable investment behavior	SIB 1	I consider environmental factors when making investment decisions	Zhang et al. (2024)
	SIB 2	I prefer companies with strong social responsibility practices	
	SIB 3	I avoid investing in firms with poor environmental or ethical records	
	SIB 4	I focus on long-term sustainable returns rather than short-term gains	
Investor financial resilience	IFR 1	I can manage my investments even during market downturns	Holmlund et al. (2017)
	IFR 2	I remain financially stable despite market fluctuations	
	IFR 3	I am confident in handling financial shocks	
	IFR 4	I can recover from investment losses over time	
Human–AI interaction competency	HAI 1	I understand how AI tools generate investment recommendations	Xiao and O’Neill (2016); Schepman and Rodway (2020)
	HAI 2	I can interpret AI-based outputs effectively	
	HAI 3	I feel comfortable using AI tools for investment decisions	
	HAI 4	I can evaluate whether AI suggestions are useful or not	

The questionnaire was in three sections: (i) questionnaire introduction and consent form, (ii) a questionnaire on the investment characteristics and demographic attributes, and (iii) measurement items. There were 24 items of six constructs, with all items on a 5-point Likert scale (strongly disagree to strongly agree), as recommended by Hair et al. (2019).

The measurement items were adapted from prior research. The questionnaire was pilot tested for clarity and reliability. No items were negatively worded.

### 3.3 Data analysis technique

The data analysis was performed using Partial Least Squares Structural Equation Modelling (PLS-SEM) with SmartPLS, as it is suitable for evaluating complex models with higher-order constructs, mediation, moderation, and non-normally distributed data (Hair et al., 2019; Sarstedt et al., 2021). The process involved two steps: measurement model and structural model assessments.

During the first stage, the measurement model was evaluated with factor loadings, composite reliability (CR) and average variance extracted (AVE). Factor loadings greater than 0.70, CR values in the range of 0.60–0.95 and AVE values greater than 0.50 were considered acceptable for reliability and convergent validity. The Fornell-Larcker criterion and the heterotrait-monotrait ratio (HTMT) were used to assess discriminant validity (Fornell and Larcker, 1981; Hair et al., 2019).

In the second stage, the structural model was evaluated in terms of path coefficients, coefficient of determination (*R*^2^), predictive relevance (*Q*^2^), and effect sizes (*f*^2^). Variance inflation factor (VIF) was used to test for collinearity. The significance of hypothesised relationships, such as mediation and moderation, was tested using bootstrapping with 5,000 resamples (Hair et al., 2019; Sarstedt et al., 2021).

## 4 Results and analysis

The results in this section show the empirical results based on PLS-SEM. Measurement model is initially tested with the aim of establishing reliability and validity and structural model is subsequently tested to test the proposed direct, mediating, and moderating relationships.

### 4.1 Demographic profile of respondents

The demographic profile of the respondents is shown in Table 2. The sample is largely comprised of males (53.6%), followed by females (46.4%), suggesting a fairly equal distribution of gender. The majority of respondents are in the age groups of 26–35 years (33.7%) and 36–45 years (28.5%), indicating that the sample is mainly young to middle-aged investors.

**Table 2:** Demographic profile of respondents.

Characteristics	Category	Frequency (*n* = 569)	Percentage (%)
Gender	Male	305	53.6
	Female	264	46.4
Age (years)	18–25	104	18.3
	26–35	192	33.7
	36–45	162	28.5
	46 or above	111	19.5
Educational qualification	SSLC	21	3.7
	HSC	97	17.0
	Graduate	289	50.8
	Postgraduate	134	23.6
	Others	28	4.9
Average monthly income (₹)	No regular income	51	9.0
	₹11,000–₹25,000	110	19.3
	₹25,001–₹50,000	162	28.5
	₹50,001–₹1,00,000	166	29.2
	₹1,00,001–₹1,50,000	59	10.4
	₹1,50,001–₹2,50,000	21	3.7
Investment experience (years)	< 1 year	82	14.4
	1–3 years	180	31.6
	4–6 years	166	29.2
	More than 7 years	141	24.8
Trading frequency	Daily	219	38.5
	Several times a week	162	28.5
	Weekly	95	16.7
	Occasionally	93	16.3
Domicile	Bengaluru	104	18.3
	Chennai	87	15.3
	Delhi	100	17.6
	Hyderabad	84	14.8
	Kolkata	78	13.7
	Mumbai	116	20.4
AI usage level	Beginner	148	26.0
	Intermediate	297	52.2
	Advanced	124	21.8

As for educational background, the majority of investors are graduates (50.8%) and post-graduates (23.6%), reflecting a highly educated investor population. In terms of income, a large proportion of the sample falls in the ₹25,001–₹1,00,000 category, reflecting the middle-income nature of the study.

Investment experience is largely centred around the 1–3 years category, suggesting a burgeoning but active investor base. Trading patterns reveal a significant number of respondents engage in frequent or daily trading. Moreover, a majority of respondents report moderate usage of AI, suggesting active engagement with AI-based trading tools.

The sample is drawn from major metropolitan cities with greater representation of Mumbai, Bengaluru, and Delhi ensuring geographical representation and representation of important urban investor segments.

### 4.2 Measurement model assessment

Measurement model was tested before the structural analysis to determine the reliability of indicators, internal consistency reliability, convergent and discriminant validity and collinearity diagnostics as per PLS-SEM guidelines (Hair et al., 2019; Sarstedt et al., 2021).

#### 4.2.1 Indicator reliability

**Figure 2:**
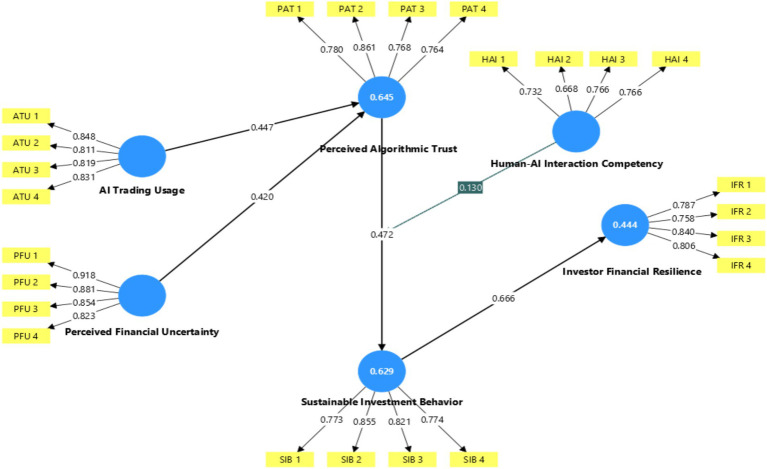
Measurement model diagram.

Indicator reliability was evaluated by observing the outer loadings of the measurement items. As shown in Figure 2 and Table 3, the indicator loadings are mostly above the recommended level of 0.70, ranging from 0.668 to 0.918, suggesting good indicator reliability. One indicator (HAI2 = 0.668) is slightly lower than the threshold but was retained because it is above the recommended minimum level of 0.60 and does not cause construct validity issues (Hair et al., 2019).

**Table 3:** Outer loadings of latent constructs.

Construct	Indicator	Outer loadings	Status
AI trading usage	ATU 1	0.848	Passed
	ATU 2	0.811	Passed
	ATU 3	0.819	Passed
	ATU 4	0.831	Passed
Human–AI interaction competency	HAI 1	0.732	Passed
	HAI 2	0.668	Passed
	HAI 3	0.766	Passed
	HAI 4	0.766	Passed
Investor financial resilience	IFR 1	0.787	Passed
	IFR 2	0.758	Passed
	IFR 3	0.84	Passed
	IFR 4	0.806	Passed
Perceived algorithmic trust	PAT 1	0.78	Passed
	PAT 2	0.861	Passed
	PAT 3	0.768	Passed
	PAT 4	0.764	Passed
Perceived financial uncertainty	PFU 1	0.918	Passed
	PFU 2	0.881	Passed
	PFU 3	0.854	Passed
	PFU 4	0.823	Passed
Sustainable investment behavior	SIB 1	0.773	Passed
	SIB 2	0.855	Passed
	SIB 3	0.821	Passed
	SIB 4	0.774	Passed

These findings demonstrate that the indicators sufficiently represent their underlying constructs, ensuring satisfactory indicator reliability. Hence, the measurement model is acceptable for the next steps of the analysis.

#### 4.2.2 Internal consistency reliability and convergent validity

To evaluate internal consistency reliability and convergent validity, we examined Cronbach’s alpha, composite reliability (CR), and average variance extracted (AVE), which are reported in Table 4. The Cronbach’s alpha values for all constructs are greater than 0.70, suggesting adequate internal consistency (Hair et al., 2019). The composite reliability (CR) also reports values greater than 0.70, indicating reliability.

**Table 4:** Reliability and convergent validity statistics.

Construct	Cronbach’s alpha	Composite reliability (rho_a)	Composite reliability (rho_c)	Average variance extracted (AVE)
AI trading usage	0.847	0.855	0.897	0.685
Human–AI interaction competency	0.72	0.731	0.823	0.538
Investor financial resilience	0.81	0.816	0.875	0.637
Perceived algorithmic trust	0.805	0.816	0.872	0.631
Perceived financial uncertainty	0.892	0.895	0.925	0.756
Sustainable investment behavior	0.82	0.821	0.881	0.651

AVE values were used to assess convergent validity. AVE values for all constructs exceed 0.50, confirming convergent validity (Fornell and Larcker, 1981; Hair et al., 2019). In summary, the measurement model exhibits acceptable internal consistency reliability and convergent validity, and is fit for structural modelling.

#### 4.2.3 Discriminant validity

The discriminant validity of the model was evaluated using the Fornell–Larcker criterion and the heterotrait-monotrait (HTMT) ratio. As shown in Table 5, the square root of the average variance extracted (AVE) for each construct is greater than the inter-construct correlations, thereby satisfying the Fornell–Larcker criterion (Fornell and Larcker, 1981).

**Table 5:** Discriminant validity assessment using the Fornell–Larcker criterion.

Construct	ATU	HAI	IFR	PAT	PFU	SIB
AI trading usage (ATU)	**0.828**					
Human–AI interaction competency (HAI)	0.618	**0.734**				
Investor financial resilience (IFR)	0.631	0.697	**0.798**			
Perceived algorithmic trust (PAT)	0.748	0.595	0.591	**0.794**		
Perceived financial uncertainty (PFU)	0.715	0.611	0.678	0.740	**0.870**	
Sustainable investment behavior (SIB)	0.665	0.69	0.666	0.708	0.698	**0.807**

Bold values indicate the square root of AVE values used to assess discriminant validity based on the Fornell–Larcker criterion.

Furthermore, HTMT values presented in Table 6 are lower than the recommended cut-off value of 0.90, also supporting discriminant validity (Henseler et al., 2016). Together, these results confirm that the constructs are empirically distinct. Overall, the measurement model exhibits satisfactory discriminant validity as per the recommended guidelines of PLS-SEM (Hair et al., 2019).

**Table 6:** HTMT ratio.

Construct	ATU	HAI	IFR	PAT	PFU	SIB	HAI × PAT
AI trading usage (ATU)							
Human–AI interaction competency (HAI)	0.816						
Investor financial resilience (IFR)	0.750	0.899					
Perceived algorithmic trust (PAT)	0.881	0.777	0.712				
Perceived financial uncertainty (PFU)	0.819	0.773	0.788	0.862			
Sustainable investment behavior (SIB)	0.788	0.861	0.806	0.858	0.811		
Human–AI interaction competency × perceived algorithmic trust (HAI × PAT)	0.330	0.333	0.219	0.233	0.260	0.102	

#### 4.2.4 Collinearity diagnostics

Multicollinearity was evaluated using variance inflation factor (VIF) values, as shown in Table 7. VIF values below 5.0, and more conservatively below 3.3 (in case of strict criteria), then there is no multicollinearity (Hair et al., 2019). The VIF values vary between 1.000 and 2.048, which are below the recommended thresholds. This suggests that there is no multicollinearity and that the path estimates are not affected by it, confirming the validity of the structural model.

**Table 7:** Collinearity assessment (VIF).

Indicators	VIF
AI trading usage → perceived algorithmic trust	2.048
Human–AI interaction competency → sustainable investment behavior	1.598
Human–AI interaction competency × perceived algorithmic trust → sustainable investment behavior	1.081
Perceived algorithmic trust → sustainable investment behavior	1.555
Perceived financial uncertainty → perceived algorithmic trust	2.048
Sustainable investment behavior → investor financial resilience	1

In addition, the full collinearity assessment approach was used as an additional robustness check for common method bias (Kock, 2015). Since all VIF values were substantially below the conservative threshold value of 3.3, common method bias is unlikely to threaten the validity of the study.

#### 4.2.5 Model fit indices

The model was assessed using the standardized root mean square residual (SRMR) and other model fit indices reported in Table 8. The SRMR values for the saturated and estimated models were 0.087 and 0.113, respectively, which are slightly above the recommended threshold value of 0.08 (Hair et al., 2019).

**Table 8:** Model fit indices.

Fit indices	Saturated model	Estimated model
SRMR	0.087	0.113
d_ULS	2.294	3.797
d_G	1.033	1.144
Chi-square	3009.460	3156.653
NFI	0.690	0.674

Additional fit indices, including d_ULS, d_G, and the normed fit index (NFI), are also reported. The NFI values fall below the conventional threshold of 0.90, and the SRMR values are slightly above the recommended cut-off value of 0.08, indicating that the overall model fit is not optimal according to covariance-based SEM standards. However, recent PLS-SEM literature emphasizes that variance-based SEM primarily focuses on prediction, explanatory power, and predictive relevance rather than strict global goodness-of-fit measures (Hair et al., 2019; Sarstedt et al., 2021). The relatively weaker fit indices may partly be attributed to the complexity of the integrated mediation–moderation framework and the exploratory nature of AI-driven behavioral modeling in emerging financial contexts.

Despite the relatively weaker global fit indices, the model demonstrates substantial explanatory power, satisfactory predictive relevance (*Q*^2^), significant structural path relationships, and acceptable measurement properties. Accordingly, the proposed model remains suitable for predictive and explanatory analysis within the PLS-SEM framework.

#### 4.2.6 Common method bias assessment

To assess the potential issue of common method bias (CMB), Harman’s single-factor test was conducted using SPSS. All measurement items were entered into an unrotated exploratory factor analysis using principal component analysis. The results indicated that the first factor accounted for 47.196% of the total variance, which is below the recommended threshold of 50% (Podsakoff et al., 2003). Therefore, common method bias is unlikely to pose a serious concern in this study.

### 4.3 Structural model assessment

**Figure 3:**
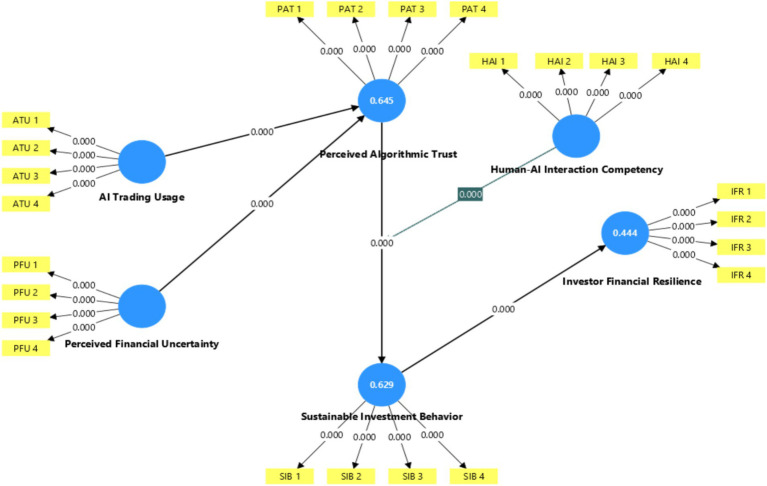
Path coefficients of the structural model.

After the adequate review of the measurement model, the structural model was reviewed to determine the hypothesized relationships between the latent constructs. The PLS-SEM evaluation of structural models pays attention to the importance and strength of path coefficients (*β*), explanatory power (*R*^2^), predictive relevance (*Q*^2^), Effect size (*f*^2^), mediation and moderation impact (Hair et al., 2019). The findings are discussed in Figure 3 and Tables 9–13.

**Table 9:** Structural path coefficients and hypothesis testing.

Hypothesis	Structural Path	*β* (path coefficient)	*t*-value	*p*-value	Hypothesis support
H1a	AI trading usage → perceived algorithmic trust	0.447	11.014	<0.001	Supported
H1b	Perceived financial uncertainty → perceived algorithmic trust	0.420	9.768	<0.001	Supported
H2	Perceived algorithmic trust → sustainable investment behavior	0.472	13.435	<0.001	Supported
H3	Sustainable investment behavior → investor financial resilience	0.666	28.258	<0.001	Supported

**Table 10:** Structural model evaluation results: *R*^2^, *Q*^2^, and *f*^2^ statistics.

Endogenous construct	*R* ^2^	*Q* ^2^	Relationship	*f* ^2^	Effect size
Perceived algorithmic trust (PAT)	0.645	0.641	ATU → PAT	0.275	Medium
			PFU → PAT	0.242	Medium
Sustainable investment behavior (SIB)	0.629	0.606	PAT → SIB	0.386	Large
			HAI → SIB	0.334	Medium
			HAI × PAT → SIB	0.045	Small
Investor financial resilience (IFR)	0.444	0.502	SIB → IFR	0.797	Large

**Table 11:** Mediation analysis results.

Hypothesis	Indirect path	Indirect effect (*β* value)	*t*-value	*p*-value	Hypothesis support
H4a	AI trading usage → perceived algorithmic trust → sustainable investment behavior	0.211	9.415	<0.001	Supported
H4b	Perceived financial uncertainty → perceived algorithmic trust → sustainable investment behavior	0.198	7.163	<0.001	Supported
H5	Perceived algorithmic trust → sustainable investment behavior → investor financial resilience	0.314	12.656	<0.001	Supported

**Table 12:** Sequential mediation analysis results.

Hypothesis	Sequential path	Indirect effect (*β* value)	*t*-value	*p*-value	Hypothesis support
H6a	AI trading usage → perceived algorithmic trust → sustainable investment behavior → investor financial resilience	0.141	8.993	<0.001	Supported
H6b	Perceived financial uncertainty → perceived algorithmic trust → sustainable investment behavior → investor financial resilience	0.132	6.997	<0.001	Supported

**Table 13:** Moderation analysis results.

Hypothesis	Moderating effect	Interaction (*β*)	*t*-value	*p*-value	Hypothesis support
H7	Human–AI interaction competency × perceived algorithmic trust → sustainable investment behavior	0.130	5.390	<0.001	Supported

#### 4.3.1 Direct effects and hypothesis testing

The structural model was evaluated using bootstrapping (5,000 resamples) to assess the significance and strength of the hypothesized relationships. The results are presented in Table 9 and illustrated in Figure 3.

The direct hypotheses (H1a, H1b, H2 and H3) are positive and significant, confirming the proposed relationships. AI trading usage has a positive impact on perceived algorithmic trust (*β* = 0.447, *t* = 11.014, *p* < 0.001), followed by perceived financial uncertainty (*β* = 0.420, *t* = 9.768, p < 0.001), underscoring the importance of both technology use and uncertainty in shaping trust.

Perceived algorithmic trust has a positive impact on sustainable investment behavior (*β* = 0.472, *t* = 13.435, *p* < 0.001), suggesting trust in AI recommendations leads to sustainable investment. Sustainable investment behavior also has a significant impact on investor financial resilience (*β* = 0.666, *t* = 28.258, *p* < 0.001), reflecting the underlying behavioral process of the model. Overall, the results support the hypothesized relationships, with sustainable investment behavior showing the strongest impact.

#### 4.3.2 Structural model evaluation: explanatory power (*R*^2^), predictive relevance (*Q*^2^), and effect sizes (*f*^2^)

We examined the coefficient of determination (*R*^2^) to measure explanatory power and the Stone–Geisser *Q*^2^ statistic to measure predictive relevance. Effect size (*f*^2^) was also tested to assess effect size of predictor constructs. According to established guidelines, *R*^2^ values of 0.25, 0.50, and 0.75 correspond to weak, moderate, and substantial explanatory power respectively, while *f*^2^ values of 0.02, 0.15, and 0.35 indicate small, medium and large effects (Hair et al., 2019). Predictive relevance is demonstrated by *Q*^2^ values greater than zero (Stone, 1974; Geisser, 1974).

Table 10 demonstrates that perceived algorithmic trust (*R*^2^ = 0.645) and sustainable investment behavior (*R*^2^ = 0.629) have substantial explanatory power, while investor financial resilience has moderate explanatory power (*R*^2^ = 0.444). All endogenous variables have strong predictive relevance (*Q*^2^ = 0.641 for PAT, 0.606 for SIB, and 0.502 for IFR).

The effect sizes show that the AI trading usage and perceived financial uncertainty exert medium effects on perceived algorithmic trust. Perceived algorithmic trust has a large effect on sustainable investment behavior, whereas Human–AI interaction competency has a medium direct effect and small moderating effect. Sustainable investment behavior has a large effect on investor financial resilience, highlighting its dominant role.

Overall, the findings show strong explanatory and predictive power with significant effect sizes.

#### 4.3.3 Mediation analysis

##### 4.3.3.1 Indirect effects

The mediation effects were calculated using bootstrapping (5,000 samples) and are shown in Table 11. All indirect effects are positive and significant (*p* < 0.001), supporting the hypotheses of mediated effects.

AI trading usage also has an indirect impact on sustainable investment via perceived algorithmic trust (*β* = 0.211, *t* = 9.415, *p* < 0.001), supporting H4a. Likewise, perceived financial uncertainty exhibits an indirect influence on sustainable investment behavior through perceived algorithmic trust (*β* = 0.198, *t* = 7.163, *p* < 0.001), highlighting the role of trust in transforming external effects into sustainable investment behavior.

Perceived algorithmic trust exerts an indirect effect on investor financial resilience via sustainable investment behavior (*β* = 0.314, *t* = 12.656, *p* < 0.001), supporting H5, suggesting that sustainable investment behavior plays a vital intermediary role.

The indirect effect via sustainable investment behavior exhibits the most significant influence. Overall, the results highlight the importance of the internal behavioral mechanisms in the model.

##### 4.3.3.2 Sequential mediation analysis

The sequential mediation effects were tested using bootstrapping (5,000 resamplings) and the findings are reported in Table 12. The two sequential indirect effects are positive and statistically significant at the 0.001 level, supporting the hypotheses.

Perceived algorithmic trust and sustainable investment behavior show a sequential mediation effect on investor financial resilience through AI trading usage (*β* = 0.141, *t* = 8.993, *p* < 0.001), supporting H6a. Perceived financial uncertainty also has a sequential impact on investor financial resilience through this mediation route (*β* = 0.132, *t* = 6.997, *p* < 0.001), confirming H6b.

These findings indicate a sequential influence of external factors on investor financial resilience via perceived algorithmic trust and sustainable investment behavior. The AI trading usage pathway has a slightly stronger effect than the other pathways.

#### 4.3.4 Moderation analysis

The moderation effect of Human–AI interaction competency on the relationship between perceived algorithmic trust and sustainable investment behavior was examined by an interaction term, as presented in Table 13. The results show a positive interaction effect (*β* = 0.130, *t* = 5.390, *p* < 0.001), thus H7 is supported.

This suggests that human–AI interaction competency enhances the association between perceived algorithmic trust and sustainable investment behavior. Investors who are more competent in interacting with AI systems are more likely to implement sustainable investment strategies when they trust the recommendations of the algorithm. The magnitude of the interaction coefficient indicates a meaningful moderating effect. The findings indicate that investor competency enhances the effectiveness of AI-assisted decision-making.

To further validate the robustness of the moderating effect, conditional direct effects were examined at different levels of Human–AI Interaction Competency. As presented in Table 14, the positive relationship between perceived algorithmic trust and sustainable investment behavior becomes progressively stronger at higher levels of Human–AI Interaction Competency.

**Table 14:** Conditional direct effects at different levels of human–AI interaction competency.

Level of human–AI interaction competency	Effect of perceived algorithmic trust → sustainable investment behavior (*β*)	*t*-value	*p*-value	Interpretation
Low competency (−1 SD)	0.342	8.788	<0.001	Weaker positive effect
Mean competency	0.472	13.637	<0.001	Moderate positive effect
High competency (+1 SD)	0.602	13.281	<0.001	Stronger positive effect

*β* = standardized path coefficient. The positive relationship between perceived algorithmic trust and sustainable investment behavior becomes progressively stronger at higher levels of human–AI interaction competency, confirming the robustness of the moderating effect.

Specifically, the effect increased from *β* = 0.342 at low competency (−1 SD) to *β* = 0.602 at high competency (+1 SD), with all effects remaining statistically significant (*p* < 0.001). These findings confirm the robustness and interpretability of the moderating effect.

### 4.4 Split-sample robustness analysis

To address concerns associated with the cross-sectional design and to assess the robustness of the findings, the sample was randomly divided into two subsamples (*n*₁ = 284; *n*₂ = 285). The structural model was re-estimated separately for both groups using PLS-SEM. As presented in Table 15, the findings revealed that the direction and statistical significance of the key structural paths remained consistent across both subsamples, supporting the stability and robustness of the proposed model.

**Table 15:** Split-sample robustness analysis.

Structural path	Group 0 (*n* = 284) *β*	*t*-value	p-value	Group 1 (*n* = 285) *β*	*t*-value	*p*-value	Robustness result
AI trading usage → perceived algorithmic trust	0.358	6.460	<0.001	0.529	9.418	<0.001	Stable
Perceived financial uncertainty → perceived algorithmic trust	0.514	9.248	<0.001	0.330	5.423	<0.001	Stable
Perceived algorithmic trust → sustainable investment behavior	0.454	8.387	<0.001	0.493	11.071	<0.001	Stable
Sustainable investment behavior → investor financial resilience	0.673	21.305	<0.001	0.661	18.980	<0.001	Stable

*β* = standardized path coefficient. All structural relationships remained positive and statistically significant across both subsamples, supporting the stability and robustness of the proposed model.

## 5 Discussion

### 5.1 Overview of the study

The study examines the role of AI-based trading on investment resilience through cognitive and behavioral processes. It uses the Stimulus-Organism-Response (S-O-R) framework to model the usage of AI trading and perceived financial uncertainty as the external stimuli, perceived trust in the algorithms as the cognitive process and sustainable investment behavior and investor financial resilience as the behavioral responses.

It uses a quantitative method, collecting data from middle-income retail investors in India and conducting Partial Least Squares Structural Equation Modeling (PLS-SEM). The model includes direct, mediation, sequential and moderation effects to represent AI trading behavior.

The analysis integrates technological, behavioral and resilience perspectives to offer a holistic view of the role of AI-enabled trading environments on responsible investors and financial resilience.

### 5.2 Key findings

The findings provide strong support for the model, highlighting the impact of AI trading environments on investor behavior and financial resilience. Perceived algorithmic trust is influenced by external factors, including perceived financial uncertainty and the use of AI-based trading strategies, implying that the relationship between investors and technology and financial conditions are vital in building algorithmic trust.

Perceived algorithmic trust is important for sustainable investment behavior, suggesting that trusting AI-driven advice helps investors to adopt more long-term and sustainable investment strategies. Sustainable investment behavior in turn aids investors in building financial resilience, implying its role in enabling financial stability during uncertain periods.

The mediation and sequential mediation results suggest that these effects are mediated by a trust-behavior process. Moreover, Human–AI interaction competency amplifies the impact of trust on behavior, suggesting the importance of user skills in using AI for financial decision-making.

### 5.3 Theoretical discussion

#### 5.3.1 AI trading and trust

Our findings demonstrate that AI trading Usage and the financial uncertainty perceived in the environment interact to impact perceived algorithmic trust, suggesting trust is formed by both user experience and the environment. These results are consistent with other studies on users’ adoption of technology and trust, which shows that trust is increased by usage of technology and uncertainty of environment (Venkatesh et al., 2012; Afroogh et al., 2024).

Increased use of AI-based tools builds confidence and familiarity with the system and increases trust in the recommendations. In contrast, uncertainty in financial markets promotes use of the AI system, since investors need advice on how to deal with the uncertain situation. This finding is consistent with behavioral research that uncertainty increases the use of analytical tools (Kahneman and Tversky, 2013).

In terms of S-O-R, AI trading Usage and perceived financial uncertainty are external stimuli that influence the state of investors’ minds via perceived trust in algorithms. This finding adds to the literature by demonstrating the combined effect of technology use and market dynamics upon trust.

#### 5.3.2 Trust as a behavioral mechanism

The results demonstrate perceived algorithmic trust is a mechanism by which technology usage influences sustainable investment. The positive impact of trust on sustainable investment behavior suggests that trust in an AI-based system helps to promote prudent and long-term investing. This is consistent with other studies that show trust helps overcome uncertainty and allows investors to trust complex systems (McKnight et al., 2011; Afroogh et al., 2024).

Theoretically, algorithmic trust is the “organism” in the S-O-R model, which translates the effects of external stimuli to behavior (Jacoby, 2002). Investors do not respond to AI systems or market factors directly, but via trust and ultimately engage in sustainable investment practices.

This is consistent with findings from the field of behavioral finance, where trust decreases cognitive effort and avoids impulsivity (Kahneman and Tversky, 2013). In AI-based decision-making, the algorithms have a “facilitative” effect on investors, promoting disciplined investing.

#### 5.3.3 Pathway to financial resilience

The research shows sustainable investment behavior is the critical pathway cognitive processes affect financial resilience. The positive association between sustainable investment behavior and investor financial resilience indicates that disciplined and long-term investment approaches increase financial resilience.

This finding supports the portfolio theory and behavioral finance research that emphasise risk diversification and prospect strategies (Kahneman and Tversky, 2013). Additionally, recent studies also suggest that Human–AI interactions result in improved financial decision-making and resilience (Xu and Cho, 2025).

In the S-O-R model, sustainable investment decisions are the “response” to cognitive processes (Jacoby, 2002). Investors concerned with sustainability are less susceptible to short-term volatility and financial shocks. This finding extends prior research by demonstrating how AI-based trust mechanisms facilitate resilience through disciplined behavior, rather than actions.

#### 5.3.4 Sequential mediation mechanism

The sequential mediation results provide evidence for a journey of how AI trading usage impacts financial resilience. Perceived trust in AI algorithms and sustainable investment mediate the influence of AI trading usage and perceived financial uncertainty on Investor financial resilience. This supports the S-O-R model by demonstrating the process by which external events are successively internalised and result in behaviors (Jacoby, 2002). Perceived algorithmic trust is the first cognitive stage, which affects how investors perceive AI recommendations, and then supports sustainable investment, and enhances financial resilience.

The process mediation implies that financial resilience is achieved through the build-up of trust and behavioral changes. This finding is consistent with dual-process theories, which highlight the interaction between cognitive processes and behaviors under uncertainty (Kahneman and Tversky, 2013).

#### 5.3.5 Moderating effect of human–AI interaction competency

The results reveal that human–AI interaction competency is a key moderating factor between perceived algorithmic trust and sustainable investment behavior. The moderation result suggests that the effect of trust on behavior increases with a higher competency of the investors in using the AI-based systems.

This study theoretically builds on the S-O-R model by adding user competency as a moderating variable between the cognitive evaluation and behavioral outcome (Jacoby, 2002). Trust in algorithms is a cognitive process, but its manifestation in investment decisions relies on investors’ skills in understanding and leveraging AI-driven insights. This result aligns with recent studies on human–AI interaction, which indicate that the user’s expertise affects trust and reliance on AI-assisted decision-making processes, ultimately supporting better financial decision-making (Afroogh et al., 2024; Xu and Cho, 2025; Hoang et al., 2026). Skilled investors can better assess the recommendations made by algorithms, not blindly follow their advice, and adequately incorporate the insights from AI into their sustainable investments.

Overall, the results suggest that the effectiveness of algorithmic trust on investment decision-making behavior depends on user competence, thereby emphasizing the need of Human–AI interaction for effective outcomes in AI-assisted financial decision-making.

### 5.4 Contributions of the study

The study makes several contributions to the fields of behavioral finance and financial technology. Firstly, it makes a theoretical contribution by extending the Stimulus-Organism-Response (S-O-R) model to AI-based trading. By positioning AI trading usage and the perceived financial uncertainty as stimuli, perceived algorithmic trust as the organism, and sustainable investment and financial resilience as responses, the research provides a comprehensive description of the interaction between technological systems and the financial environment in shaping investment decision-making. In particular, the study’s focus on perceived algorithmic trust as an important mechanism adds to the existing body of knowledge by highlighting its role in translating AI-based information into actions.

Second, the study makes a methodological contribution by employing Partial Least Squares Structural Equation Modeling (PLS-SEM) to test a model with mediation, sequential mediation and moderation. This all-encompassing view captures the context-specific, multi-step nature of AI-based financial decision-making and offers a more complete picture of investment practices.

From a computational and AI perspective, the study contributes to the emerging literature on intelligent financial decision-support systems by explaining how investors interact with AI-driven trading platforms. AI-based trading environments increasingly rely on predictive analytics, algorithmic recommendation systems, and real-time financial data processing to support investment decisions. Although the present study primarily focuses on behavioral and cognitive mechanisms rather than algorithm development, the findings contribute to AI-finance research by demonstrating how perceived algorithmic trust and Human–AI interaction competency shape the effective utilization of explainable AI-enabled financial decision-support systems in retail investing environments.

Third, the study offers a contextual contribution by looking at financial decision-making of middle-income retail investors in India using AI-based trading platforms. This offers valuable insights into how technological adoption and financial uncertainty influence investment decision-making in emerging financial markets.

### 5.5 Managerial and practice implications

This investigation has implications for investors, financial service firms and technology providers in AI-facilitated financial markets. For retail investors, effective use of AI trading systems requires them to understand the AI system. Investors can improve their financial literacy and AI literacy to support decision-making, prevent over-reliance on AI, and encourage long-term investment.

The insights also suggest that financial service providers need to develop user-friendly AI systems. Transparency on the reasoning behind AI-based recommendations, risk management and decision-making strategies can promote trust and avoid impulsive investment behavior. Behavioral nudges embedded within investment applications may encourage responsible investment practices. For AI providers, AI-based financial technologies should be designed to encourage sustainable investment behavior rather than speculative trading. Making the technology more understandable, usable and reliable can increase trust and facilitate effective investment decisions.

In summary, the effective use of AI in financial markets depends on user experience and skills to support investment decision-making.

### 5.6 Policy implications

The study offers insights for regulating AI-based trading platforms, particularly in terms of transparency, investor protection, and responsible AI governance. Providing greater transparency and explainability of algorithmic recommendations can benefit investors’ decision-making processes and minimize over-reliance on automated systems. Greater disclosure regarding how AI-generated recommendations is developed may help investors better understand the limitations and risks associated with algorithmic decision-support systems.

Regulatory bodies might also mandate that there is a risk disclaimer and behavioral safety in trading applications to shield new traders and limit impulsive trading. Policymakers should continue to promote explainable AI standards and open algorithmic governance structures to boost investor trust on AI-driven financial services. There is also a need for regulations that encourage financial and digital literacy to ensure that investors can better understand and apply AI-generated financial data. Improving the ability of human–AI interaction competency can lead to better decision-making and risk management in AI-driven trading systems.

Finally, a balanced policy approach towards regulating AI in the financial markets is needed, which ensures transparency of the technology, responsible governance of AI, and investor capacity to adopt sustainable and responsible investment practices.

### 5.7 Limitations and future research

The research has a few limitations that can be addressed in future research. The study is cross-sectional in nature and does not permit causal inferences; longitudinal or experimental studies may provide deeper insights into the evolution of investors’ behavior. The self-reported measures used in this study might suffer from response bias; the use of actual investor behavioral or trading data would improve the validity of the results. In addition, although the model demonstrated satisfactory explanatory and predictive capability, the global fit indices (SRMR and NFI) did not fully meet conventional covariance-based SEM thresholds. Therefore, the findings should be interpreted with appropriate caution. Future studies may refine the measurement model and explore alternative model specifications to improve overall model fit.

The focus of the study on middle-income retail investors in India may limit the generalizability. Cross-validating the model with different investor categories and regions would strengthen the model’s validity. The model examines human–AI interaction competency as a boundary condition, but other factors such as the transparency of algorithms, interface design and investor expertise can also be investigated. Furthermore, human–AI interaction competency was assessed using self-reported measures, which may not fully capture objective technological competency. Future research may incorporate objective indicators such as digital literacy, investment expertise, or behavioral trading measures to further validate the moderating mechanism. Another important direction for future research is the evolution of trust in AI-driven environments, particularly across different market conditions and technological environments.

## 6 Conclusion

This study examined the effect of AI trading usage on investors’ financial resilience via a cognitive-behavioral framework. The proposed framework, based on the Stimulus-Organism-Response (S-O-R) model, defined the AI trading usage and perceived financial uncertainty as stimuli, perceived algorithmic trust as the cognitive process, and sustainable investment behavior and investor’s financial resilience as responses.

The results provide strong empirical support for the proposed framework. The AI trading usage and perceived financial uncertainty have a positive impact on perceived algorithmic trust, which indicates that both technology usage and market uncertainty influence the trust of investors in the algorithmic system. Perceived algorithmic trust also increases sustainable investment behavior, implying that trust in the algorithmic system leads to sustainable investment behavior.

In addition, sustainable investment behavior supports investor financial stability, suggesting its role in promoting stability in times of uncertainty. The results of mediation and sequential mediation indicate the effect of AI-based trading on investor’s financial performance is mediated through a process of trust and subsequent behavioral reactions. The moderating role of Human–AI interaction competency also implies trust in AI-based trading is effective when investors’ competency to use AI-based information.

Taken together, this research proposes a sequential link between AI usage, trust, sustainable investment and financial resilience in the emerging market. These insights suggest that the effectiveness of AI in financial markets is not exclusively dependent on technology but also on human competency and behavior, offering insights for future AI-integration in financial markets.

## Data Availability

The raw data supporting the conclusions of this article will be made available by the authors, without undue reservation.
